# Economically sustainable shade design for feedlot cattle

**DOI:** 10.3389/fvets.2023.1110671

**Published:** 2023-01-25

**Authors:** Alex S. C. Maia, Gustavo A. B. Moura, Vinicius F. C. Fonsêca, Kifle G. Gebremedhin, Hugo M. Milan, Marcos Chiquitelli Neto, Bruno R. Simão, Victor Paschoal Consentino Campanelli, Rodrigo Dias Lauritano Pacheco

**Affiliations:** ^1^Innovation in Thermal Comfort and Animal Welfare (Inobio-Manera), Animal Biometeorology Laboratory, São Paulo State University, Jaboticabal, SP, Brazil; ^2^Brain Function Research Group, School of Physiology, University of the Witwatersrand, Johannesburg, South Africa; ^3^Department of Biological and Environmental Engineering, Cornell University, Ithaca, NY, United States; ^4^Innovation in Thermal Comfort and Animal Welfare (Inobio-Manera), Animal Biometeorology Laboratory, São Paulo State University, Ilha Solteira, SP, Brazil; ^5^Agro-Pastoril Paschoal Campanelli, Research Center, Altair, São Paulo, Brazil

**Keywords:** animal welfare, heat load, cattle, shading, profitability, sustainable intensification

## Abstract

Provision of shade reduces radiant heat load on feedlot cattle, thus reducing demand of water and energy for thermoregulation. While the positive effects of shade on animal welfare are widely known, the literature lacks data on the magnitude of its economic impacts. In this study, we propose the concept of novel shade design to prove that a correctly oriented and dimensioned roof structure, which optimizes shade to be displaced within the pens, motivates cattle to seek shade, protect them from short-wave solar radiation, and is resilient to counteract weather adverse conditions. The beneficial outcome is improvement in animal welfare and productive performance, as well as increments on financial return and sustainability. To attest these benefits, eight hundred *B. indicus* × *Bos taurus* bulls were randomly assigned in pens with or without shade from a galvanized steel-roof structure. Performance data (e.g., dry matter intake, body weight gain, feed efficiency and hot carcass weight) and heat stress indicators (e.g., subcutaneous temperature, body-surface temperature, respiratory rate and water intake) were assessed along the study period. The economic outcomes derived from shade implementation were determined using the net present value. Meteorological variables were also monitored every 1 min, and grouped in a thermal comfort index for feedlot cattle, the *InComfort Index* (InCI). The shade structure efficiently reduced radiant heat load on cattle in pens with shade. According to the classification of the InCI, during very hot days (InCI > 0.6; around noon with mean solar radiation above 800 W m^−2^ and mean air temperature above 33°C), greater proportion (80%) of animals in shaded pens were using shade. Under such circumstances, cattle in shade had water intake reduced by 3.4 L per animal, body temperature was lower by 5°C, subcutaneous temperature was lower by 1°C and respiration rate was lower by 10 breaths min^−1^ compared to animals in pens without shade (*P* = 0.0001). Although dry matter intake was similar (*P* = 0.6805), cattle in pens with shade had higher average daily gain reflected in a heavier hot carcass weight (8 kg animal^−1^; *P* = 0.0002). Considering an initial investment of $90 per animal to build a structure that lasts 15 years, the expected payback time is four finishing cycles (~110 days per cycle). In conclusion, this study confirms that the proposed novel shade design is economically profitable, improves performance, and enhances animal welfare.

## 1. Introduction

Artificial shade is in the forefront of environmental modification to mitigate the negative impacts of heat stress and to improve welfare of beef cattle, particularly in tropical environments where animals face high levels of short-wave solar radiation (1, 2). The benefits of shade for animals come from reducing radiant heat gain (3, 4), body heat storage (5), evaporative cooling through panting and sweating (6–9) and by increasing the frequency of beneficial behaviors (e.g., lying down, ruminating, and playing (10–12).

Although the positive wellbeing and behavioral effects of providing shade for animals are unquestionable, their economic benefits are still inconclusive. Some studies have found positive outcomes on animal performance and economic gains (13, 14), others did not (10, 15). These economic uncertainties could perhaps be explained by the various shade structures and experimental designs tested in previous investigations. For example, use of 30 vs. 80%-blockage shade cloth, experiments with different animal categories (heifers, steers, and bulls), and different levels of heat load experienced by cattle (16). Some experiences of Brazilian beef producers were reported using shade cloth, mostly due to its lower cost of implementation over other shade materials (15, 17). However, limitation of this type of shade infrastructure lies on poor durability and life-span (18), especially if installed at locations with heavy windy and rainy conditions (19). The uncertainty on the economic return of shade implementation may therefore explain why beef producers are still reluctant on to the idea of providing artificial shades, e.g., < 20% of feedlot in Brazil and US provide artificial shades (20–22).

In this study, we propose a novel concept of shade that could be used in tropical areas. It is a design that combines rectangular pens with a shade structure mounted in a north-south orientation. This design makes shade to be projected within the pens, motivates shading-seeking behavior of cattle, and efficiently protects them against short-wave solar radiation, while improves animal welfare, increases productivity performance, and offers an interesting 1–2 year payback time. To attest these benefits, 800 steers were randomly assigned in shaded and unshaded pens. The animals and the environment were monitored to (1) describe the thermal environment experienced by the animals, (2) determine the impact of shading on heat stress indicators, (3) assess the impacts of shading on dry matter intake, water intake, average daily gain, and hot carcass weight, and (4) provide analyses concerning economic outcomes of the shading structure. To the best of our knowledge, this is the first robust study to show the economic benefits of shade in feedlot cattle under commercial conditions of Brazil, where close to seven million cattle are kept in feedlots without shade (20). This issue is of great importance because Brazil is located in a tropical region where solar irradiance throughout the year is high and relatively constant, a climatic factor that poses great challenge to the welfare of livestock (4).

## 2. Material and methods

### 2.1. Location and design of the shading structure

**Figure 1:**
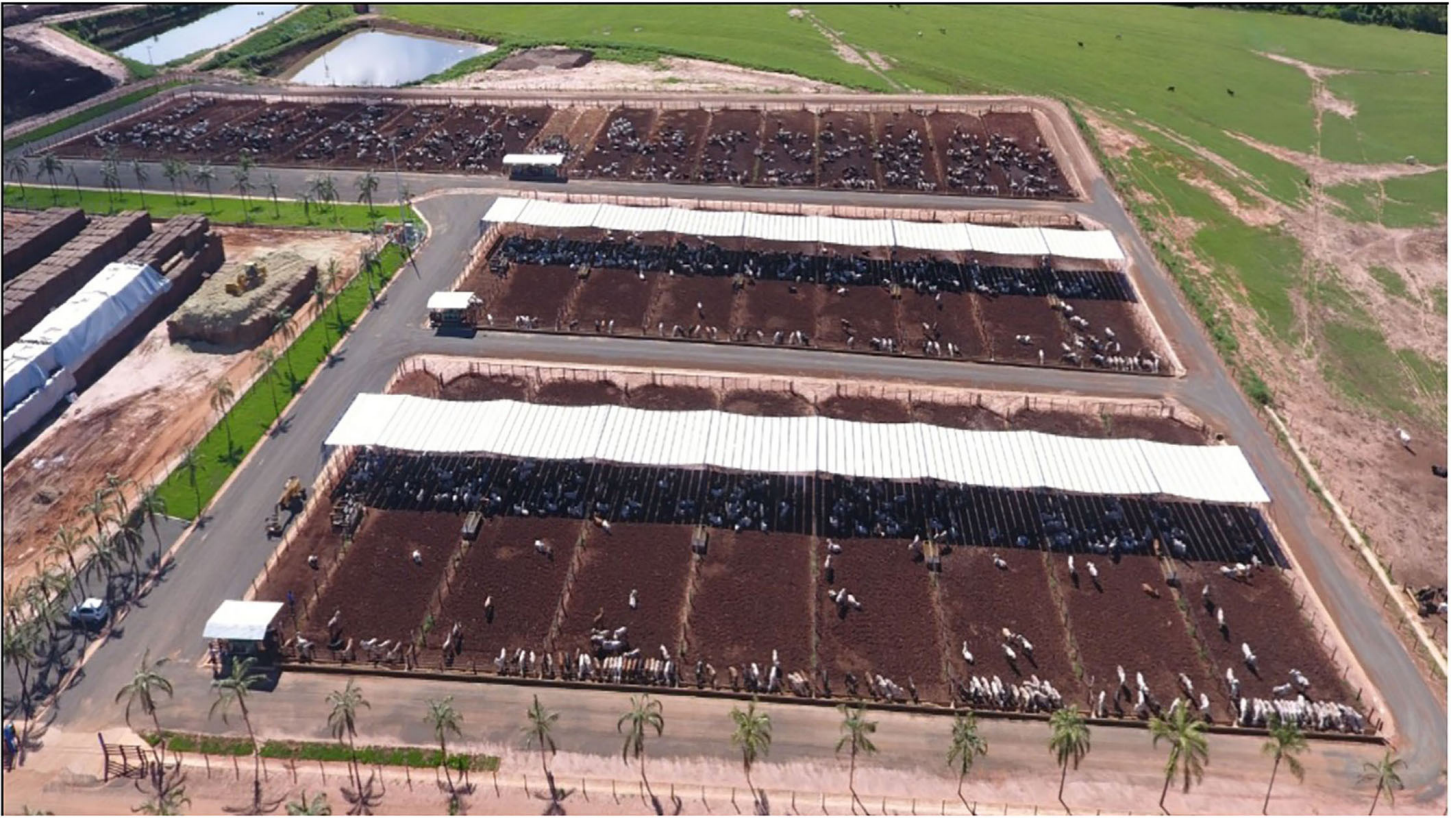
Experimental facilities of the commercial cattle feedlot in Altair, São Paulo, Brazil (20° 31' 25” S; 49° 03' 32” W).

**Figure 2:**
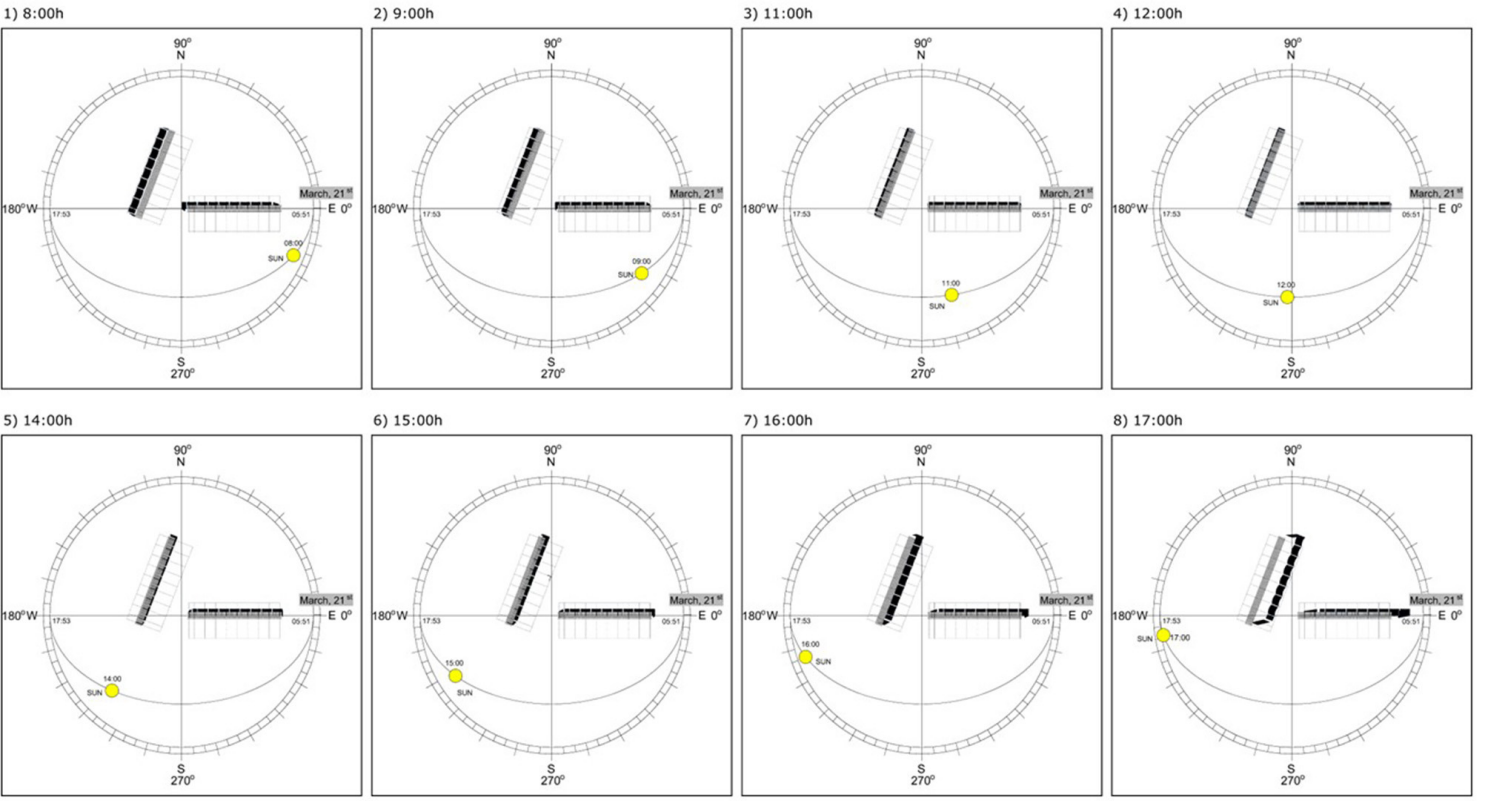
Layout of the shade displacement (from 08:00 to 17:00 h) of the shade structure at 18° of North-South orientation. The shade displacement is also simulated for East-West orientation. These simulations were made considering the latitude of the experimental site, and for the day 120 (Julian day, march, 21) of the year (2022). The space filled in black represents shade projected.

The study was performed from November 2019 to March 2020 at the experimental facilities of a commercial feedlot in Altair, São Paulo, Brazil (Agro-Pastoril Paschoal Campanelli Research Center, 20°S latitude, altitude of 557 m) (Figure 1). Overall, climate classification according to Köppen-Geiger is B^2^, with precipitation rate above 1200 mm year^−1^ occurring at summer months (From November to February), and dry winters (From May to July). The novel concept of shade design used in this study comes from a shade structure that optimizes maximum availability and displacement of shade within pens, blockage against solar radiation, resilience to weather adverse conditions, and ease of daily management operations. We proposed rectangular pens with 15 × 50 m (width × length) instead of square pens, and the shade structure installed on the north-south fence line. This design favors a moving shade from east to west within the pen, and motivates shade-seeking cattle to follow the shade, which results in reduced wet areas because of congregation of animals (Figure 2). Additional benefit of this design is that the shade structure on the fence line eliminates barriers to the equipment used to clean out the pens.

**Figure 3:**
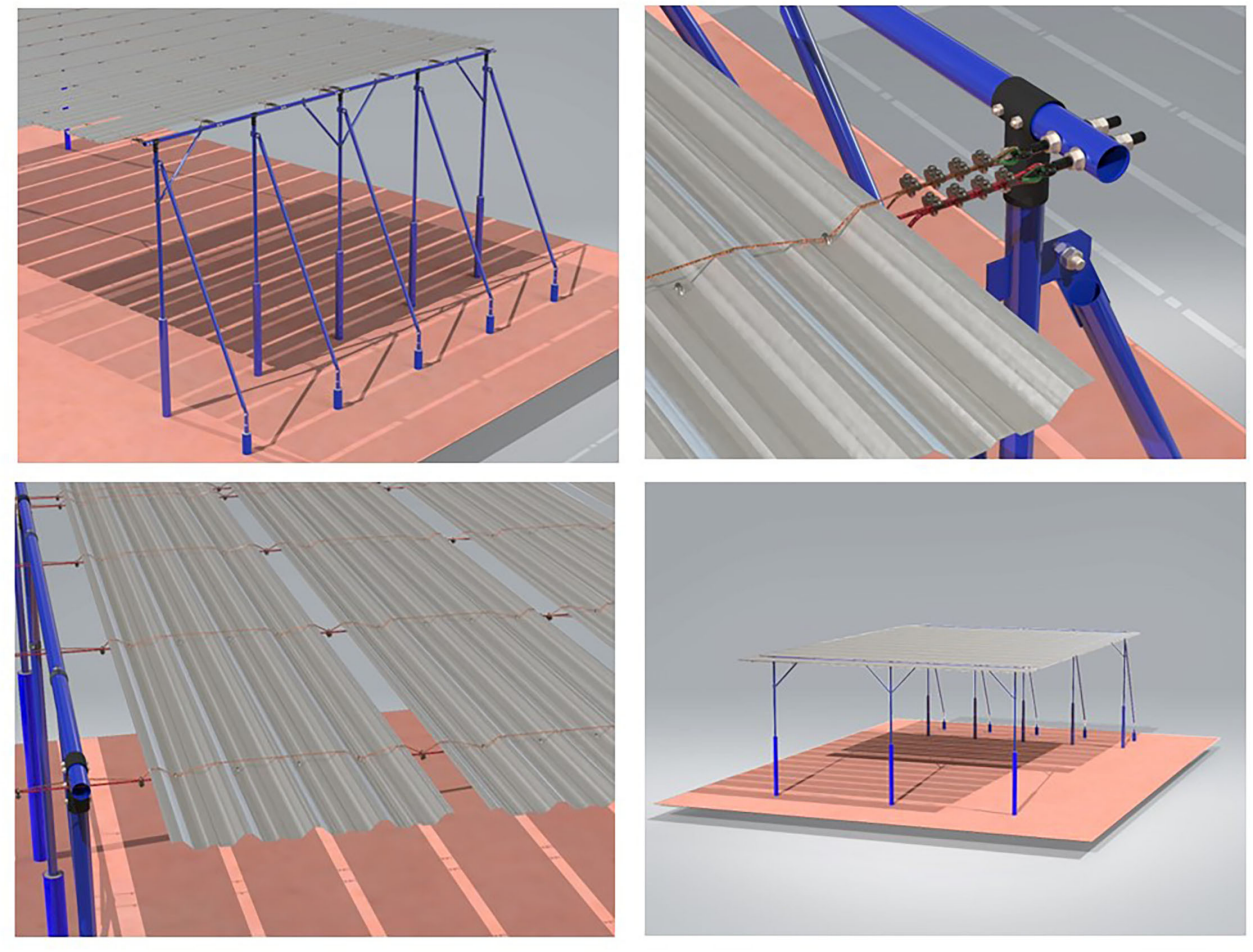
Principal components of the shade structure.

The shade structure was made from galvanized coated aluminum/zinc/silicon (55% × 43.5% × 1.5%) steel-roof structure (Galvalume; Companhia Siderúrgica Nacional, CSN). The anchors were made of steel instead of wood because the cost of steel is the same as that of wood in Brazil. The frame assembly was made of tubular beams which is easier to assemble (Figure 3). Pillars anchored with polyvinyl chloride (PVC) and concrete provide protection against corrosion. The feedlot environment is very corrosive because of high concentration of urine and feces. The height of the roof was 5 m and consisted of parallel tiers mounted 10 cm apart tied with double steel cables. This design reduces tension and gives more flexibility to the shade structure during windy weather. Moreover, the shade area that is lost by the openings is minimal.

### 2.2. Experimental design

All animal procedures were approved by the Animal Ethics Committee of the São Paulo State University (Protocol number 016339/19). A total of 800 *B. indicus* × *Bos taurus*, predominantly Nellore bulls were used in the study. Approximately 2 weeks before the beginning of the study, the animals were treated with anthelmintic medication (1 mL 20 kg BW of 10% fenbendazole, MSD Saúde Animal, São Paulo, Brazil), immunized against bovine respiratory disease (1 mL 45 kg of BW; MSD Saúde Animal, São Paulo, Brazil), and clostridia (5 ml animal^−1^; Valée S/A Produtos Veterinários, Montes Claros, Brazil).

Sixteen soil-surfaced pens were used, eight of them had the novel shade design that provided 3.0 m^2^ of shade floor area/animal. The 800 bulls were equally divided between pens with shade, and pens without shade (50 animals pen^−1^). Each bull was provided with 15 m^2^ space, 0.30 cm of concrete bunk space, and 3.0 × 0.8 × 0.25 m (length × width × height) water trough. The bulls were assigned in blocks by their fasted initial body weight (BW_*i*_ ± SEM) (Block 1 = 305 ± 0.10 kg; block 2 = 337.80 ± 0.10 kg; block 3 = 375.20 ± 0.01 kg; and block 4 = 413 ± 0.10 kg of BW_*i*_), and weekly harvested (one block per week) in the order of heavier to lighter block. Furthermore, bulls were phenotypically characterized by a trained observer on the basis of muscularity, structure, body length, hip and wither height. They were then grouped into five different phenotypes: Nellore, Black Angus x Nellore, crossbred (Zebu breeds, mostly Nellore), crossbred (European dairy breeds), and crossbred (European beef breeds).

### 2.3. Nutritional management

Prior to the experiment, bulls were purchased from nine different locations (average transportation distance of 480 km) and allocated in three 16 ha pastures of *Cynodon ssp*., equipped with feeding bunk. During this phase, all animals received pre-experimental (maintenance) diet (Table 1), for at least 15 days, in order to reestablish physiological and ruminal conditions. During the experimental period, bulls were fed with adaptation (days 1 to 14), growing (days 15 to 35) and finishing diets (block 4 = days 36 to 109; block 3 = days 36 to 114, block 2: days 36 to 119; and block 1: days 36 to 124), formulated to meet or exceed an average daily gain (ADG, kg day^−1^) of 1.5 kg (Table 2) (24). Bulls were fed twice daily (08:30h and 15:00h), and visual bunk score calls were daily recorded at 06:45h (25). Diet dry matter adjustments of ingredients were then performed on a daily basis using a Koster Moisture Tester (Model D, Koster Crop Tester Inc. Medina Ohio, USA). Feed refusals of every pen were daily collected and weighted before the first meal. Treatment composite samples of ration, refusals and feedstuffs were daily and weekly collected (respectively) and then frozen at −20°C to determine dry matter intake (DMI, kg animal^−1^ day^−1^) and for chemical analyses. All the samples from diets were dried at 55°C in a forced-air oven for 72 h for DM determination. Dried samples were grounded with a Wiley-type mill (1 mm screen, MA-680, Marconi Ltda, Piracicaba, São Paulo, Brazil) and analyzed for ash (method 924.05) (26), NDF (27), CP (28) and EE (method 920.85) (29).

**Table 1:** Values used in the economic analyses of the shade structure.

**Variables (Running costs)**	**Treatments (pens)**	
	**Shaded**	**Unshaded**
Carcass value, R$ 15 kg^−1^	55.00	55.00
Cost of shade implemented, R$ animal^−1^	90.00	0
Capital cost per feedlot cycle, %	5	5
Funrural tax, %	2.1	2.1
Earned income tax, %	10	10
Animal transport guide fees, R$ animal^−1^	2.60	2.60
**Benefits**		
Hot carcass weight, kg animal^−1^	326.51	317.97
Carcass value at slaughter, R$ animal^−1^	55.00	55.00
Lifespan of the shade structure, years	15	-

All monetary values are in USD$.

**Table 2:** Ingredients and nutrients of feed diets used in the shaded and unshaded pens.

**Feed ingredient (%, DM basis)**	**Diets**			
	**Pre-exp**	**Adaptation**	**Growing**	**Finishing**
High moisture corn	6.85	8.50	10.65	14.95
Citrus pulp	9.86	27.40	35.80	36.45
Cottonseed meal	-	8.90	8.70	10.30
Soybean meal	15.07	15.07	12.60	9.35
Sugar cane bagasse	20.55	5.50	5.80	3.75
Sugar cane silage	30.82	-	-	-
Corn silage	-	19.20	13.00	11.20
Protected fat^b^	-	-	2.30	3.10
Urea	0.91	0.90	0.95	1.00
Molasses	13.70	12.35	7.85	7.50
Tracemineral supplement^a^	3.15	2.30	2.40	2.45
**Nutrients, %**				
Dry matter	45.00	62.00	68.00	69.00
Crude protein	12.80	16.00	14.60	13.70
Degradable intake protein (% of crude protein)	79.00	80.00	77.00	79.00
Non-fibrous carbohydrates	42.00	47.00	48.00	49.00
Total digestible nutrients	75.00	86.00	85.00	88.00
peNDF^d^	21.00	19.00	17.00	16.00
Ca	0.93	1.25	1.45	1.47
P	0.42	0.45	0.40	0.40
NEm, Mcal/kg^c^	1.49	1.85	1.85	1.90
NEg, Mcal/kg^c^	0.90	1.25	1.20	1.30

a Calcium 165.0 g/kg, phosphorus 23.0 g/kg, cobalt 25.0 mg/kg, copper 420.0 mg/kg, sodium 40.0 g/kg, sulfur 14.0 g/kg, iodine 25.0 mg/kg, magnesium 15.0 g/kg, manganese 810.0 mg/kg, selenium 15.0 mg/kg, zinc 1,500.0 mg/kg, iron 0 mg/kg, vitamin A 72,000 IU/kg, vitamin D3 14,370 IU/kg, vitamin E 500 IU/kg, monensin 714.0 mg/kg, virginiamycin 714.0 mg/kg.

b Calcium soap, approximately 86% of fatty acids and 14% of calcium.

c Estimated by L.R.NS.

d Fraction of the fiber that stimulates chewing activity and maintains a healthy rumen environment by combining the chemical and physical properties of feeds (23).

#### 2.2.1. Water intake and performance data

Water was freely available. Each water trough was equipped with hydrometers to determine daily water intake (DWI; m^3^ animal^−1^ day^−1^). Hydrometer readings were taken every morning. The average daily water intake (DWI, m^3^ animal^−1^ day^−1^), dry matter intake (DMI, kg animal^−1^ day^−1^), average daily gain (ADG, kg animal^−1^ day^−1^), and conversion (FC, feed: gain ratio) were determined for each pen. The DWI was determined by measuring water flow to the water troughs as follows:


$$\begin{array}{c} DWI_{i}=\frac{{\left({\text{H}}_{2}\text{O}\right)}_{ij}}{N_{ij}} \end{array}$$

Where, H_2_O is the amount of water intake in the *i*^*th*^ ordinal day of the experiment in the *j*^th^ pen (*j* = 1,…,16) and *N* is the number of animals in the *i*^*th*^ ordinal day of the experiment in the *j*^th^ pen. The DMI was determined based on the difference of the offered and refused feed, as follows:


$$\begin{array}{c} DMI_{i}=\frac{{\left(f_{o}-f_{r}\right)}_{ij}}{N_{ij}} \end{array}$$

Where, *f*_*o*_ is the amount of dry matter offered (kg day^−1^), while *f*_*r*_ is the amount of dry matter refused collected daily at the feed bunk. The ADG were calculated based on fasted initial body weight (B*W*_*i*_, kg animal^−1^; 16 h of feed and water withdrawal) and fasted final body weight (B*W*_*f*_, kg animal^−1^, 16 h of feed and water withdrawal) as follows,


$$\begin{array}{c} \text{ADG }=\frac{{\text{BW}}_{f\;}-\;{\text{BW}}_{i}}{n} \end{array}$$

Where, *n* is the total number of days on feeding. The BW_*i*_ and BW_*f*_ were obtained using a scale that was regularly calibrated with 450 kg standard-weight. The feed conversion (FC) was calculated as follows,


$$\begin{array}{c} \text{FC}=\;\frac{DMI}{\text{ADG}} \end{array}$$

After reaching their expected BW_*f*_, the animals were transported (330 km) to a commercial packing plant, and hot carcass weight (HCW, kg animal^−1^) was obtained after complete evisceration and remotion of kidney, pelvic and heart fat of the carcass.

#### 2.2.2. Meteorological data

Solar irradiance (R_S_, W m^−2^; CMP-22, Kipp and Zonen, Delft, Netherlands; spectral range = 0.3–3.6 μm), ultraviolet solar radiation (U_V_, W m^−2^; spectral range = 0.28–0.4 μm), air temperature (T_A_,°C; range = −40 to +70, accuracy ± 0.1°C), black-globe temperature in the sun (T_Gsun_,°C; accuracy ± 0.1°C), relative humidity (R_H_, %; accuracy ± 3 %), wind speed (W_S_, m s^−1^; accuracy ± 0.44, m s^−1^) and daily precipitation (P, mm h^−1^), were all continuously recorded every minute with a portable weather station (WS-18 model 110, Nova Lynk, Auburn, CA, USA) placed near (~50 m) the pens with shade and without shade. Temperature sensors were also placed inside the pens and water troughs to better characterize the microclimate experienced by the bullocks in the shaded and unshaded pens. These measurements were recorded every 5 min.

**Figure 4:**
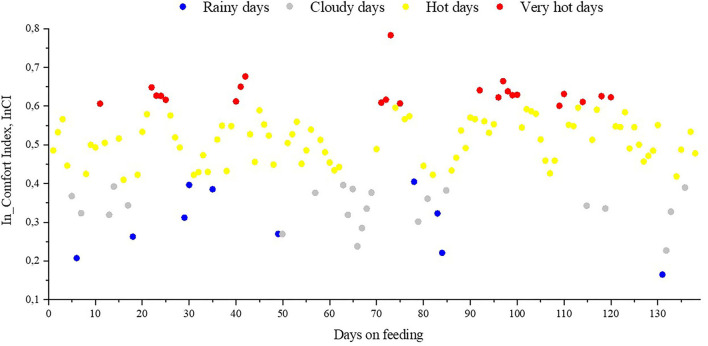
Daily mean values of the InComfort Index (InCI) over the days on feeding. Rainy days (0 ≤ InCI ≥ 1, and precipitation rate above 20 mm d^−1^); cloudy days (0 ≤ InCI ≥ 0.4); hot days (0.4 ≤ InCI ≥ 0.6); very hot days (0.6 ≤ InCI ≥ 1). There were 8 days classified as rainy, 24 as cloudy, 83 as hot, and 23 as very hot over the days on feeding. Refer to Table 3 and Figure 5 for details of meteorological conditions experience by feedlot cattle in each class of days.

A set of six black-globe devices were placed in two shaded pens (Figure 4), and three black globes were placed in three unshaded pens, positioned two meters above the ground surface. Miniature data loggers (i-bottom DS1925L, Maxim Integrated, Sao Jose, US; size = 0.60 × 1.70 cm, height × diameter; accuracy ± 0.5°C) were inserted inside globes for measuring black-globe temperature. The black-globe devices in pens with shade were placed underneath the roof (T_Gshade1_,°C) and exposed to clear sky (T_Gshade2_, T_Gshade3_,°C). Three temperature sensors (i-buttom) were previously waxed (Sasol wax, GmbH D-20457) and placed inside the water troughs to obtain water temperature (°C). The ground surface temperature underneath the shade and in the full sun were periodically measured with an infrared thermal camera (FLIR SC660; Wilsonville, USA; temperature range = −40°C to 1500°C; spectral range = 7.5 to 13 μm; adjustable emissivity range = 0.1 to 1; resolution ± 0.04°C; accuracy ± 1°C).

#### 2.2.3. Thermal stress indicators

Subcutaneous temperature (T_SC_,°C), respiratory rate (R_R_, breaths min^−1^), and body surface temperature (T_S_,°C) were measured both in bulls housed in shaded and unshaded pens. Behavior of bulls in the shaded pens were also observed. Ten days before the experimental period, miniature implantable bio-loggers (i-buttom DS1922L, Maxim Integrated, Sao Jose, US; size = 0.60 × 1.70 cm, height × diameter; accuracy ± 0.06°C) were surgically implanted in forty animals, twenty in the shaded pens, and the other twenty in the pens without shade. Before the implantation, all the loggers were calibrated at 2°C increments between 30 and 42°C in a thermally insulated box against a highly-accurate thermocouple (Type K; temperature range = −40 to 1300°C; accuracy ± 0.2°C). Logging and storage of T_SC_ data was set to measure every 2 min during the experimental period.

The R_R_ and T_S_ were recorded every 30 min from 08:00 to 17:00 h, on 80 animals (40 in the shaded and another 40 in the unshaded pens) during 20 days. Respiration rate was observed by five observers positioned outside the pens (~10 m apart from the animals) by visually counting the flank movements of the bulls. Body surface temperature was measured using an infrared thermal camera (FLIR SC660; emissivity = 0.98) scanning the dorsal right side of the animals at a distance of 3.0 m. For each bull, the T_S_ was considered as a mean of the scanned dorsal right side, from the scapular to rump region. Images were analyzed using FLIR Thermal Studio. Number of bulls using shade or at feed bunk was periodically monitored by using direct, focal and instantaneous samplings every 15 min, from 08:00 to 17:00 h. A total of 1500 observations from 250 animals were scanned along 20 days during adaptation, growing and finishing phase. Shade use was recorded whenever the head or one of the hooves of an animal was underneath the shade area. If not, it was considered as exposed to solar radiation. Feed bunk use was recorded whenever an animal was at standing position close (~0.5 m) to the feed bunk.

### 2.3. Economic outcomes

To determine the economic outcomes and the payback time of the proposed shade design, we calculated the net present value (NPV) as


$$\begin{array}{c} NPV=\;\left[\frac{CF_{1}}{{\left(1+r\right)}^{1}}+\frac{CF_{2}}{{\left(1+r\right)}^{2}}+\frac{CF_{1}}{{\left(1+r\right)}^{3}}+\frac{CF_{n}}{{\left(1+r\right)}^{n}}\right]-\Pi_{0} \end{array}$$

Where, Π_0_ is the initial investment of the shade structure ($), *FC*_(*n*)_ is net cash flow for a given period of days; *r* is the cost of the capital, and *n* is the number of days of each feedlot cycle (~110 days). We estimated the initial investment of the artificial shade structure in USD$ 90.00 animal^−1^ (USD$ 30.00 per m^2^ of projected shade, assuming 3 m^2^ animal^−1^).

### 2.4. Statistical analyses

#### 2.4.1. Heat load experienced by feedlot cattle

Principal component analyses (30–34) were used to observe dissimilarities of the days of feeding for the meteorological conditions (T_A_, H_R_, R_S_, U, W_S_ and T_G_) experienced by the animals. Principal components were obtained by computing eigenvalues (λ_i_) and its respective eigenvectors $e_{i}^{,}=\left[e_{i1}\;e_{i2}\;e_{i3}\right]$ of the data correlation matrix. Bi-dimensional representation of the multidimensional set was created by using scores of the first (*PCA*_1*j*_ = *e*_11_*T*_*A*_ + *e*_12_*H*_*R*_ + *e*_13_*R*_*S*_ + *e*_14_*U* + *e*_15_*W*_*S*_ + *e*_16_*T*_*G*_), and second principal component (*PCA*_2*j*_ = *e*_21_*T*_*A*_ + *e*_22_*H*_*R*_ + *e*_23_*R*_*S*_ + *e*_24_*U* + *e*_25_*W*_*S*_ + *e*_26_*T*_*G*_). All principal components were used in order to develop an environmental index, the *InComfort Index* (InCI) based on the Membership Function Value Analysis:


$$\begin{array}{c} \text{InCI}=\;\overset{\text{n}}{\underset{\text{i}=1}{\sum }}\left[\text{R}\left({\text{λ}}_{\text{i}}\right)\text{W}\left({\text{e}}_{\text{i}}\right)\right] \end{array}$$

Where, n is the number of principal components and InCI is the weighted membership value calculated with principal components for each day linked with its meteorological condition and level of heat stress experienced by the animals. The *R*(λ_*i*_) is given by


$$\begin{array}{c} \text{R}\left({\text{λ}}_{\text{i}}\right)=\frac{{\text{λ}}_{\text{i}}-{\text{λ}}_{\text{i}\left(\text{min}\right)}}{{\text{λ}}_{\text{i}\left(\text{max}\right)}-{\text{λ}}_{\text{i}\left(\text{min}\right)}} \end{array}$$

Where, λ_i_ is the value of i^th^ principal component, λ_i(min)_ and λ_i(max)_ are the maximum and minimum values of i^th^ principal component, respectively. The W(e_i_) is given by


$$\begin{array}{c} \text{W}\left({\text{e}}_{\text{i}}\right)={\text{e}}_{\text{i}}/\overset{\text{n}}{\underset{\text{i}=1}{\sum }}{\text{e}}_{\text{i}} \end{array}$$

Where, *W*(*e*_*i*_) is the weight of the i^th^ principal component among all the principal components selected for evaluating level of heat stress experienced by animals on i^th^ day, and e_i_ is the contribution rate of the i^th^ principal component. Based on daily water intake and respiratory rate, the InCI were grouped into four classes: rainy days, when the mean is 0 ≤ InCI ≤ 1, and precipitation rate above 20 mm day^−1^; cloudy days, when the mean is 0 ≤ InCI ≤ 0.4; hot days, when 0.4 < InCI ≤ 0.6; and very hot days, when the mean is 0.6 < InCI ≤ 1.

#### 2.4.2. Confirmatory models

In this study, we investigated the effects of the following independent variables on cattle performance and heat stress responses: shade vs. no shade, weight block, coat color (black vs. light-colored cattle), time of day, heat load experienced during the days of feeding and association between them. Confirmatory models were then fitted by applying conventional statistical techniques through mixed model based on Generalized Least Squares (GLS) using the Statistical Analysis System [SAS Institute, Version 8; (35)]. Because of the repeated nature of the data (e.g., days on feeding and time of day), the covariance structure of the model must be chosen carefully (36). Different covariance structures were tested (compound symmetry, first-order auto regression, Toeplitz, first-order ante-dependence and others) and the best covariance structure was chosen based on the Akaike's information criterion (AIC), AIC corrected (AICC), and Bayesian information criterion (BIC). To choose the best fitted models that predict dry matter intake (a), daily water intake (b) average daily gain, and (c) hot carcass weight (d) the following independent variables were considered:


[a, b] $$\begin{array}{l} Y_{ijkl}=\mu +S_{\text{i}}+B_{\text{j}}+SB_{\text{ij}}+H_{\text{k}}+SH_{\text{ik}}+SBH_{\text{ijk}}+D_{\text{l}}{\left(SBH\right)}_{\text{ijk}}\\ \;+\varepsilon_{\text{ijklm}} \end{array}$$

[c, d] $$\begin{array}{l} Y_{ijkl}=\mu +S_{\text{i}}+SP_{\text{ij}}+B_{\text{k}}+SB_{\text{ik}}+C_{\text{l}}{\left(SB\right)}_{\text{ij}}+\varepsilon_{\text{ijklm}} \end{array}$$

Where, *Y*_*ijkl*_ is the *n*th measurement of the dependent variable; S is the fixed term of the *i*th shade treatment (S = shade and unshaded pens); B is the fixed effect of the *j*th class of initial fasted body weight [B = block 1 (305 kg), block 2 (337.80 kg), block 3 (375.20 kg), block 4 (413 kg)]; SB is the interaction between the *i*th shade treatment and *j*th class of body weight; H is the fixed effect of the *k*th class of the InComfort index (H = rainy, cloudy, hot and very hot days); SH is the interaction between the *j*th shade treatment and *k*th class of the InComfort index; SBH is the interaction between the *i*th shade treatment, *j*th class of body weight and *k*th class of the InComfort index; and D is the fixed effect of the *l*th days on feeding (D = 1,…,124 days) within the interaction between *i*th shade treatment, *j*th class of body weight and *k*th class of the InComfort index. Models c and d had SP as interaction between *i*th shade treatment and *j*th phenotypic trait of cattle, and C is the fixed effect of the *l*th corral pen within the interaction between *i*th shade treatment and *k*th class of body weight shade treatment. The μ is the parametric mean and ε_ijklm_ is the residual term. For subcutaneous temperature (e), body surface (f), and respiratory rate (g) the best fitted models were:


[e] $$\begin{array}{l} Y_{ijklm}=\mu +S_{\text{i}}+C_{\text{j}}+SC_{\text{ij}}+H_{\text{k}}+D_{\text{l}}{\left(H\right)}_{\text{k}}+T_{\text{m}}\\ \;+SCHT_{\text{ijkm}}+\varepsilon_{\text{ijklm}} \end{array}$$

[f, g, h] $$\begin{array}{l} Y_{ijk}=\mu +S_{\text{i}}+C_{\text{j}}+SCT_{\text{ijk}}+\varepsilon_{\text{ijkl}} \end{array}$$

Where, S is the fixed term of the *i*th shade treatment; C is the fixed effect of the *j*th coat color (C = Dark and light colored cattle); SC is the interaction between the *i*th shade treatment and *j*th coat color; H is the fixed effect of the *k*th class of the InComfort index; D is the fixed effect of the *l*th days on feeding within *k*th class of the thermal comfort; T is the fixed effect of the *m*th time of the day (T = 1,…, 24 h); and SCHT is the interaction between *i*th shade treatment, *j*th coat color, *k*th class of the InComfort index and *m*th time of the day. For models f, g, and h, SCT is the interaction between *i*th shade treatment, *j*th coat color and *k*th classes of solar irradiance. ε_ijklm_ and ε_ijkl_ are residual terms. Behavioral data were analyzed using nonparametric regression analyses through the Generalized Additive Models (GAM Procedure), by fitting air temperature, black-globe temperature, solar irradiance, and wind speed as independent variables.

## 3. Results and discussion

We confirmed the hypothesis that the proposed novel shade design not only benefits cattle comfort, but is also economically sustainable. Three main findings support this hypothesis: First, based on the radiant temperatures taken within the shade and full sun, the structure with shade efficiently reduced radiant heat load on the animals, especially on very hot days (**Figure 7**). During these days heat stress indicators were significantly reduced compared to bulls without access to shade. Overall, the values were 5°C lower in body-surface temperature, 1°C lower in subcutaneous temperature, 10 breaths min^−1^ lower for respiration rate, and 3.4 L animal^−1^ lower for water intake (**Figures 6**, **7**, **9**, **10**; *P* = 0.0001). Even though dry matter intake was similar (*P* = 0.6805) for the bulls in shade and no shade (**Table 4**), those in pen with shade presented better feed conversion (*P* = 0.0004) and heavier hot carcass weight (*P* = 0.0002; **Figure 11**). The increment on hot carcass weight of the bulls in pens with shade was sufficient reason to make the proposed shade design economically viable. The payback time is expected to be within four feeding cycles (**Figure 12**).

**Figure 5:**
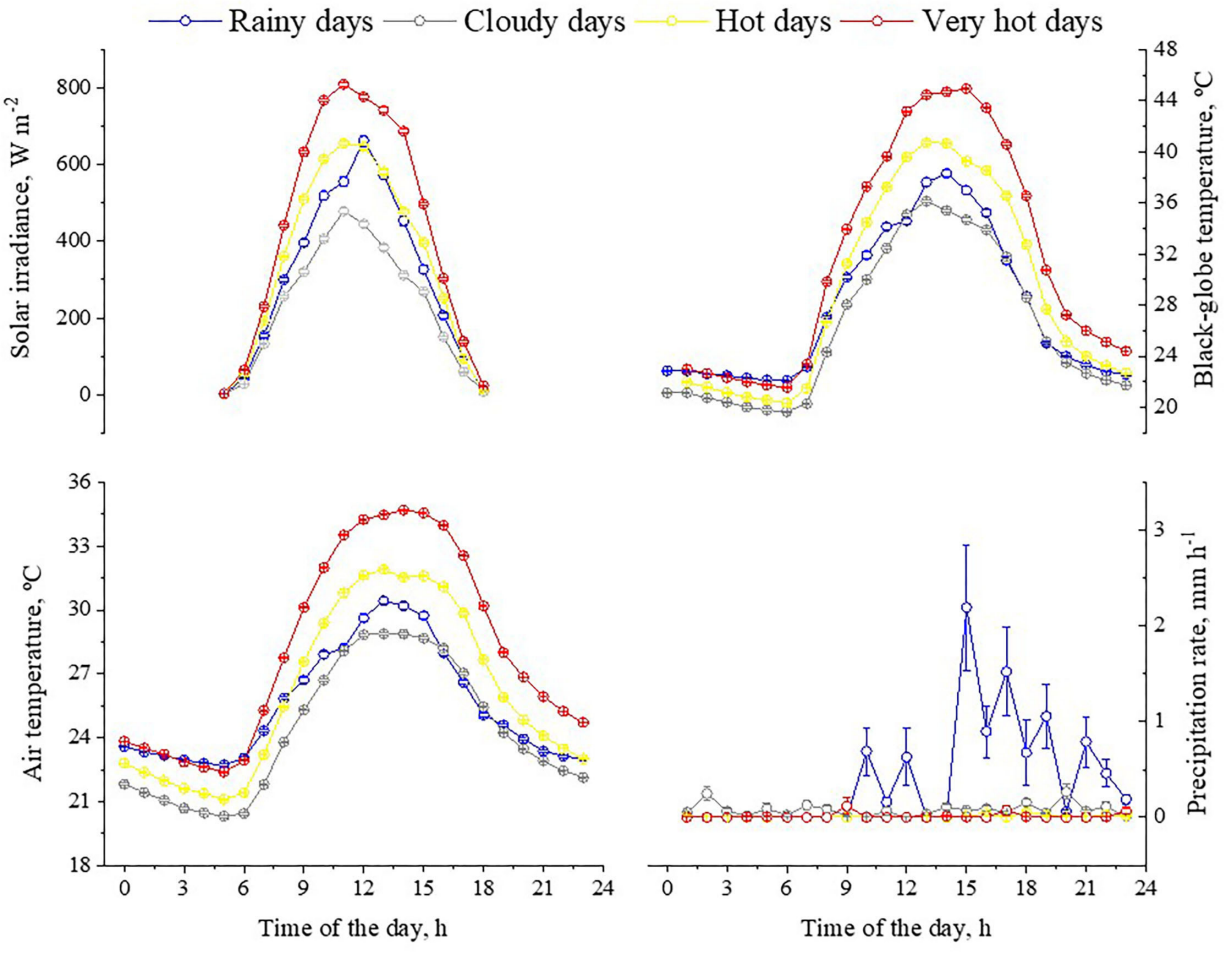
Least square (±SEM) means of the meteorological variables measured during the experimental period in accordance to the classes of the InComfort Index (InCI). Classes of the InCI: Rainy days (0 ≤ InCI ≥ 1, and precipitation rate above 20 mm d^−1^); cloudy days (0 ≤ InCI ≥ 0.4); hot days (0.4 ≤ InCI ≥ 0.6); very hot days (0.6 ≤ InCI ≥ 1).

**Figure 6:**
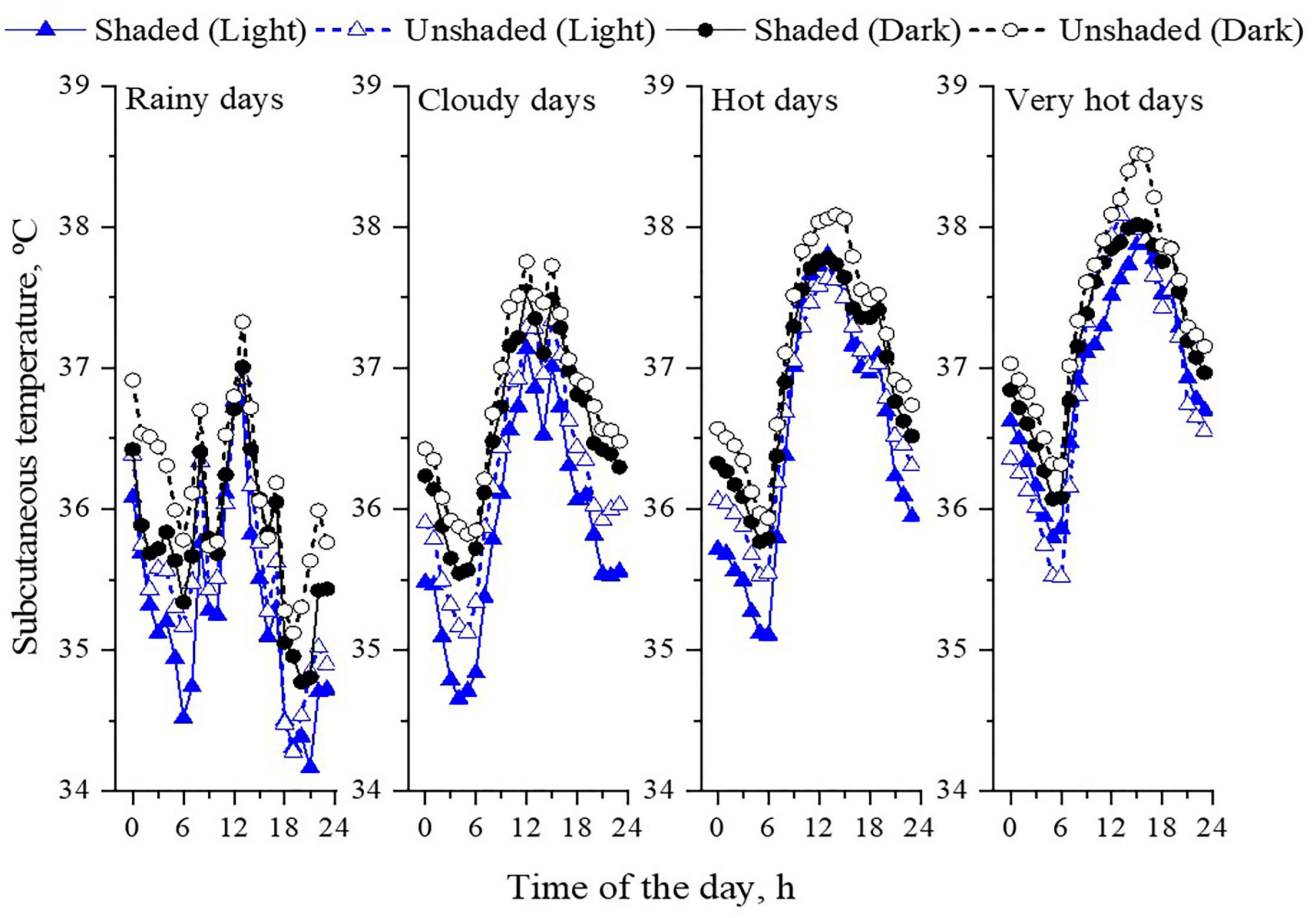
Daily pattern of subcutaneous temperature in light (predominantly Nellore) and black colored (*Bos indicus* x *Bos taurus*), shaded and unshaded bulls in accordance to the classes of the InComfort Index (InCI). Classes of the InCI: Rainy days (0 ≤ InCI ≥ 1, and precipitation rate above 20 mm d^−1^); cloudy days (0 ≤ InCI ≥ 0.4); hot days (0.4 ≤ InCI ≥ 0.6); very hot days (0.6 ≤ InCI ≥ 1).

The meteorological conditions experienced by the feedlot cattle were characterized by a single variable, an environmental index for beef cattle (InComfort Index, InCI). The variable accounted for the combined effects of solar irradiance, black-globe and air temperature, humidity, wind speed, and precipitation rate. Feeding days were grouped into four classes (Table 3; Figures 4, 5). During the experimental period, the bullocks were exposed for 100 days to hot and very hot conditions (e.g., 23 very hot days and 83 hot days). During these days, from 10:00 to 15:00h, mean solar radiation exceeded 600 W m^−2^, black-globe temperature was above 40°C, and air temperature was above 30°C. In addition to hot conditions, bulls were exposed to 23 cloudy days, during which the mean solar irradiance remained below 600 W m^−2^, and mean air temperature was below 30°C. Bulls were also exposed to eight rainy days, during which the mean precipitation rate ranged from 0 to 0.25 mm h^−1^. Based on our previous study with similar animals, feedlot cattle were outside their zone of least thermoregulation during the days classified as hot and very hot (8, 37–40). Indeed, bulls stored more heat during hot and very hot days compared to the cooler days (i.e., rainy and cloudy days), especially the animals with dark hair coat and with no access to shade (Figure 6).

**Table 3:** Meteorological variables (mean, min, and max) during the study period according to environmental index (InComfort, InCI).

**Meteorological variables**	**Classes of InCI** ^**†**^			
	**Rainy days (*****n*** = **8)**	**Cloudy days (*****n*** = **24)**	**Hot days (*****n*** = **83)**	**Very hot days (*****n*** = **23)**
Air temperature,°C	23.80 (18.0–37.5)	24.5 (14.0–37.7)	26.0 (15.7–38.5)	28.0 (18.0–41.0)
*Black globe temperature,°C	27.5 (16.8–48.2)	26.0 (12.3–48.3)	28.8 (14.5–53.5)	31.5 (16.8–55.0)
Relative humidity, %	80.0 (35–100)	75.5 (23.0–97.0)	70.0 (21.0–98.0)	66.0 (25.0–98)
Solar irradiance, W m^−2^	265.75 (0–1100)	264.60 (0–1090)	488 (0–1199)	635 (0–1250)
Daily precipitation, mm d^−1^	43 (21–106)	2.12 (0–14)	1.76 (0–18)	2.05 (0–4.5)
Wind speed, m s^−1^	2.0 (0–15)	3.0 (0–14)	3.0 (0–15)	3.0 (0–10)
Water temperature,°C	25 (23–30)	26 (22.8–28)	28 (23–30)	29 (22.9–31)

^*^Black globe temperature taken in the shade; ^†^Rainy days (0 ≤ InCI ≥ 1, and precipitation rate above 20 mm d^−1^); cloudy days (0 ≤ InCI ≥ 0.4); hot days (0.4 ≤ InCI ≥ 0.6); very hot days (0.6 ≤ InCI ≥ 1).

**Figure 7:**
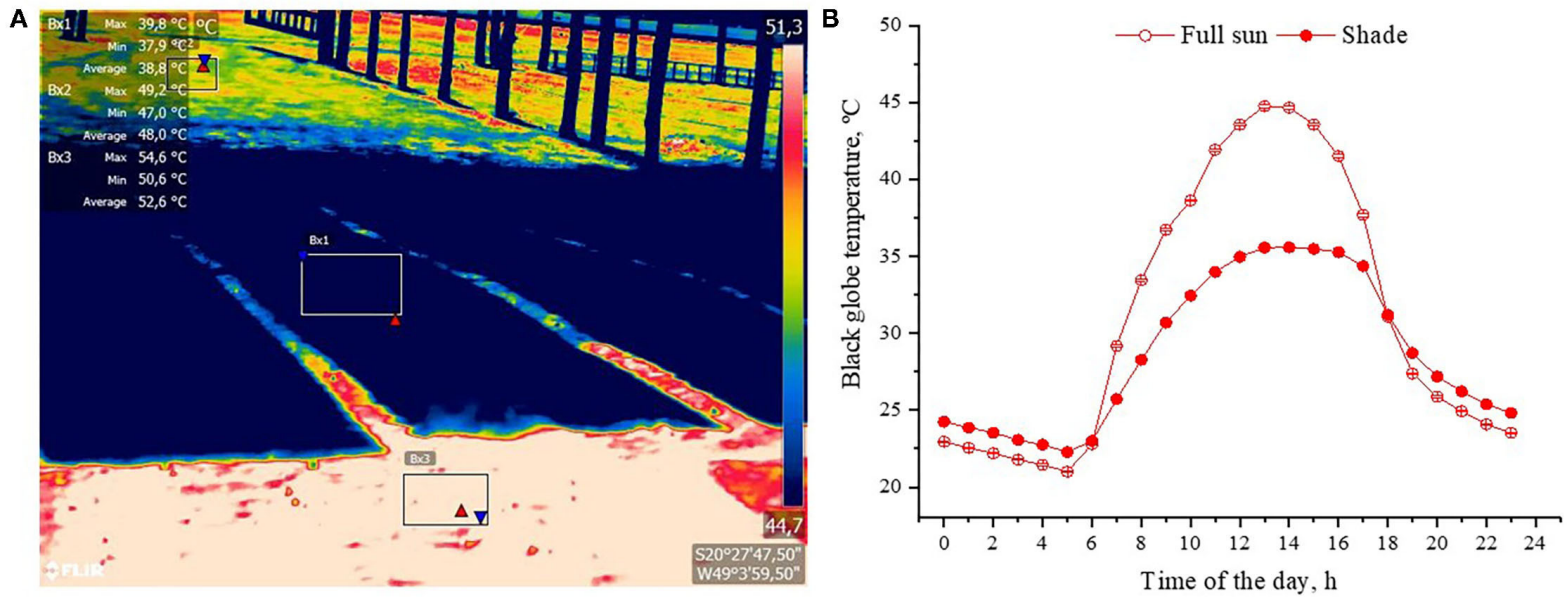
Radiant heat load experienced by feedlot cattle; **(A)** thermogram of a shaded and unshaded ground surface. Meteorological conditions that this thermal image was captured were: Air temperature = 35°C; solar irradiance = 850 W m^−2^; **(B)** Least square means (±SEM) of the black globe temperature taken in full sun and shade in days classified as very hot (0.6 ≤ InCI ≥ 1).

Our study well attested that shade alleviated heat stress responses of cattle, mostly due to the abatement of radiant heat gain through two main sources: From direct and diffuse short-wave solar radiation, where on hottest times of hot and very hot days, by seeking shade, cattle could avoid levels of solar irradiance as much as 1200 W m^−2^ (Table 3). Animals with dark hair coat absorb almost three times more solar radiation than those with light hair (4, 5, 41). This difference in solar load explains why the body surface temperature of dark bulls is higher compared to light-haired cattle (**Figure 9**). Animals in shade also received less long-wave radiation emitted from the shaded surface. During the hottest time of the very hot days, the radiant temperature of the shaded ground surface was 15°C lower than the unshaded areas, a difference that represents 100 W m^−2^ less long-wave radiation being emitted from the ground surface (Figure 7). Bulls in pens with shade also experience less heat gain by conduction when lying down on shaded surfaces.

**Figure 8:**
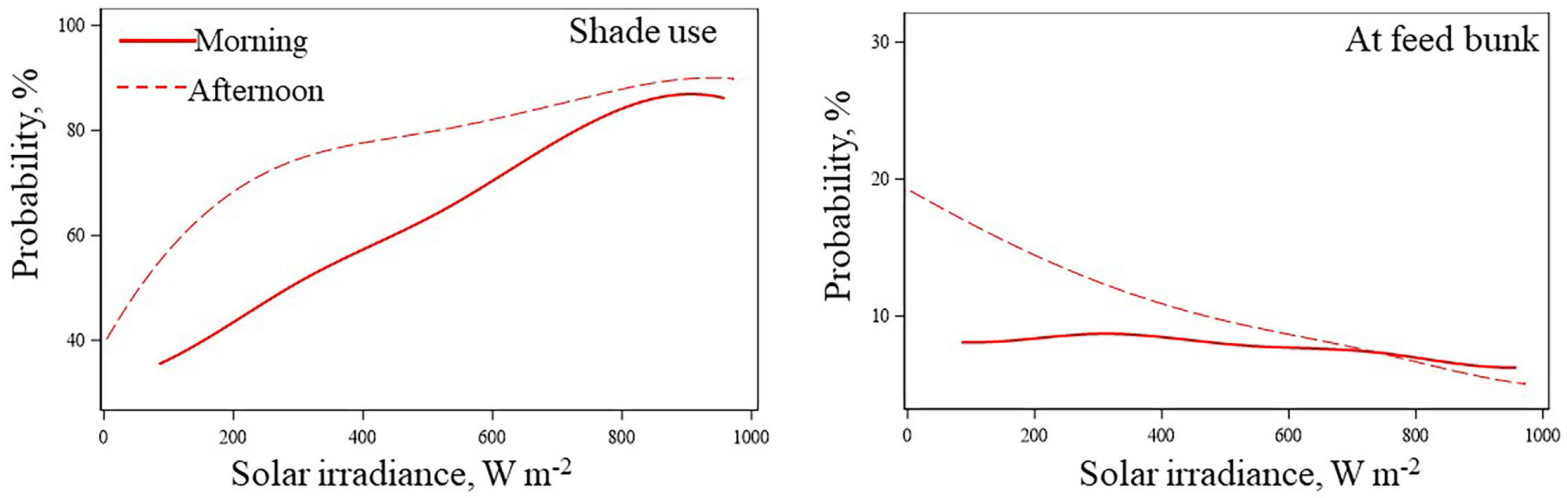
Estimated probabilities as a function of solar irradiance for bulls to be in shade or at the feed bunk.

**Figure 9:**
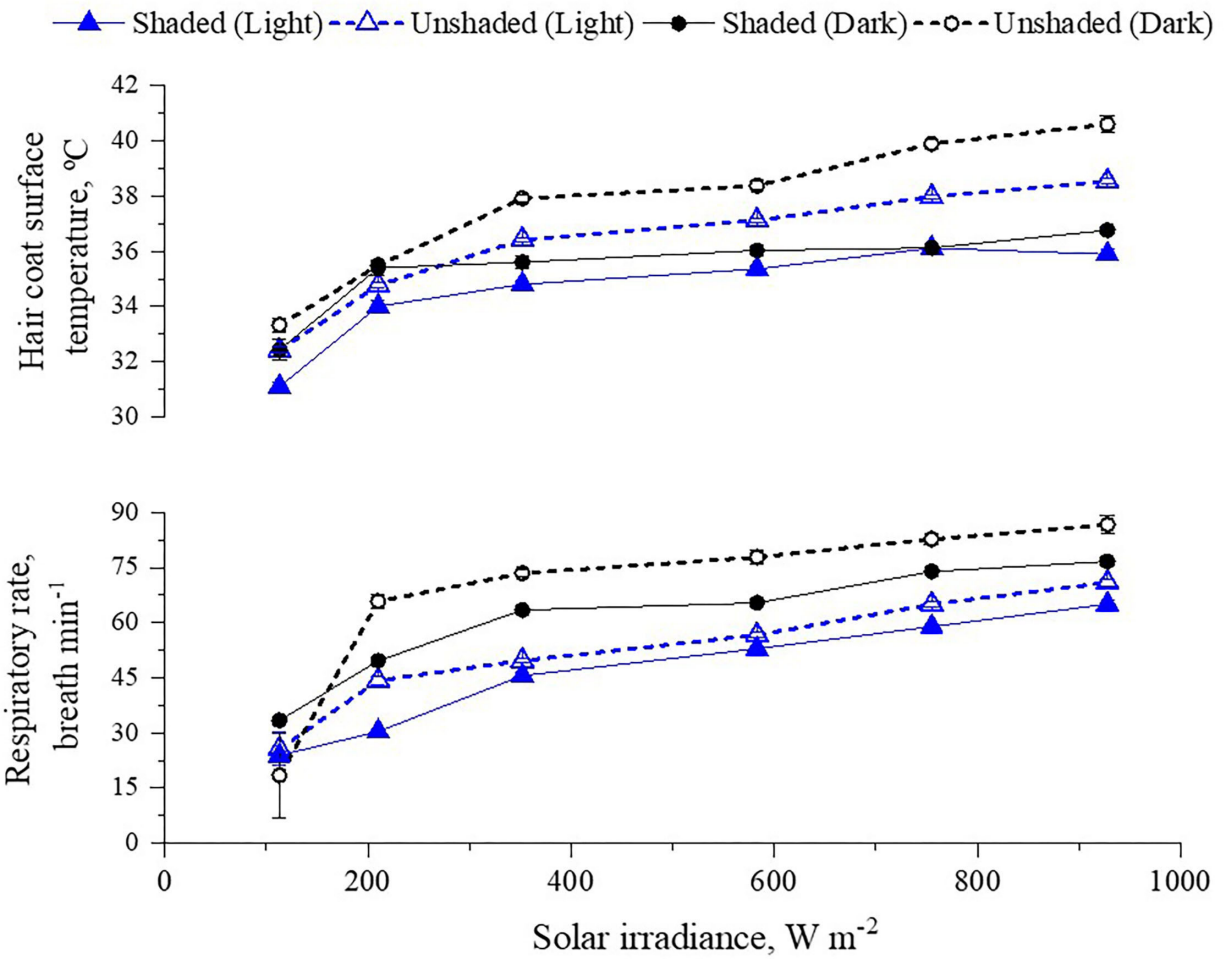
Least square means (±SEM) of hair coat surface temperature and respiratory rate of shaded and unshaded light- and dark-haired cattle according to the classes of solar irradiance.

Maximum shade utilization of cattle occurred when levels of solar irradiance exceeded 800 W m^−2^, either in the morning or afternoon (Figure 8; probability higher than 80%), a level of thermal radiation that also coincided with the fewer probabilities for bulls to be at feed bunk. Bulls in shade had their respiratory rate reduced and overall were less peripherally vasodilated than bulls in pens without shade as heat load increased (Figures 6, 9). The reduction was higher for cattle with dark hair coat. Similarly, Lees et al. (42) attested that both *B. taurus* dark hair coat (Black Angus) and *B. indicus* light hair coat cattle (Brahman) sought shade as the solar load increased, and panted less when they were in the shade. Considering that increments on respiratory rate and peripheral vasodilatation are positively correlated with mass and heat transfer through respiratory and cutaneous surface (8, 43), bulls in shade trigger less heat dissipation to maintain thermal equilibrium compared to bullocks with no access to shade.

**Figure 10:**
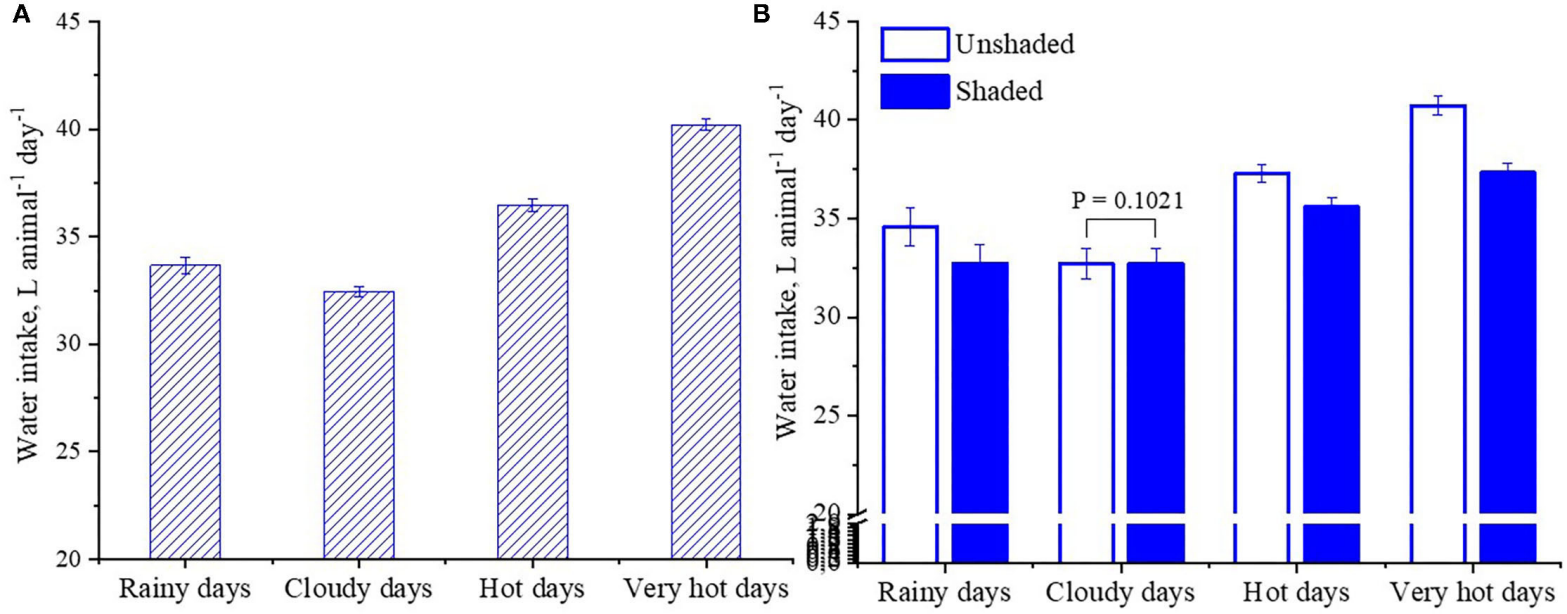
Least square means (±SEM) of daily water intake of feedlot cattle according to the classes of the InComfort Index (InCI). Classes of the InCI: Rainy days (0 ≤ InCI ≥ 1, and precipitation rate above 20 mm d^−1^); cloudy days (0 ≤ InCI ≥ 0.4); hot days (0.4 ≤ InCI ≥ 0.6); very hot days (0.6 ≤ InCI ≥ 1). **(A)** Fixed effect of the InCI. **(B)** Effects of shade treatments within the InCI.

When cattle are exposed to hot conditions and are unable to access shade, they can sustain high rates of evaporative water loss to maintain their thermal equilibrium, provided that body fluids can be replaced timeously (44), a physiological adjustment that increases requirements for water turnover. In this study, it was observed that bulls in pens with and without shade increased water intake during hotter days (Figure 10). However, bulls in pens without shade presented greater water intake than bullocks in shade (36.1 vs. 34.9 ± 0.19 L animal d^−1^). The increase was greater during the very hot days (40.73 vs. 37.30 ± 0.47; Figure 10). (45) also reported greater water intake for *B. taurus* cattle housed in pens without shade compared to cattle in shade (53 vs. 49 L animal^−1^ d^−1^), particularly when exposed to hot days. In this study, using mean difference of 3.43 L animal^−1^ d^−1^ for cattle in pens without shade vs. cattle in pens with shade during 23 very hot days, and extrapolating to 100 days in a year with similar meteorological conditions and for 20 000 animals, the use of shade would potentially save 6860 m^3^ of clean water. The saving emphasizes the effectiveness of using shade to alleviate heat stress of cattle and reduce water consumption.

Use of the proposed novel shade design increased efficiency in dry matter intake. Both groups of cattle in shade and no shade ate less during hotter and rainy days. Bulls may decrease intake during hotter days as a tentative mechanism to reduce heat generation by metabolism (2). During rainy days, we speculate that cattle consume less due to an overall reduction in activity and due to soaked feed in the bunks. The dry matter intake was not statistically different between cattle with or without shade availability (Table 4), an average of 11.45 and 11.51 ± 0.17 kg animal^−1^ d^−1^, for shaded and unshaded cattle, respectively. However, cattle in pens with shade presented better feed conversion (*P* = 0.0389), which was nearly improved by 4.5 % (6.83 vs. 7.15 ± 0.10) as well as increased average daily gain (*P* = 0.0004) by 5% (1.657 vs. 1565 ± 0.01 kg animal^−1^ d^−1^) compared to cattle housed in pens without shade (Table 4). This result shows that use of shade may reduce energy requirements for maintenance of feedlot cattle in tropical conditions.

**Table 4:** Performance data (least square means ±SEM) of shaded and unshaded cattle.

**Item**	**Treatments (Pens)**		**SEM**	***P*-value**
	**Unshaded**	**Shaded**		
Initial body weight, kg	358.78	358.88	0.6895	0.9146
Final body weight, kg	562.96	574.55	2.1034	0.0001
Average daily gain, kg d^−1^	1.565	1.657	0.0182	0.0004
Dry matter intake, kg d^−1^	11.43	11.51	0.1730	0.6805
Dry matter intake, %	2.36	2.33	0.0383	0.6204
Feed conversion	7.15	6.83	0.1036	0.0389
Hot carcass weight, kg	317.97	326.51	1.5917	0.0002
Dressing percentage	56.62	56.87	0.1091	0.1110

**Figure 11:**
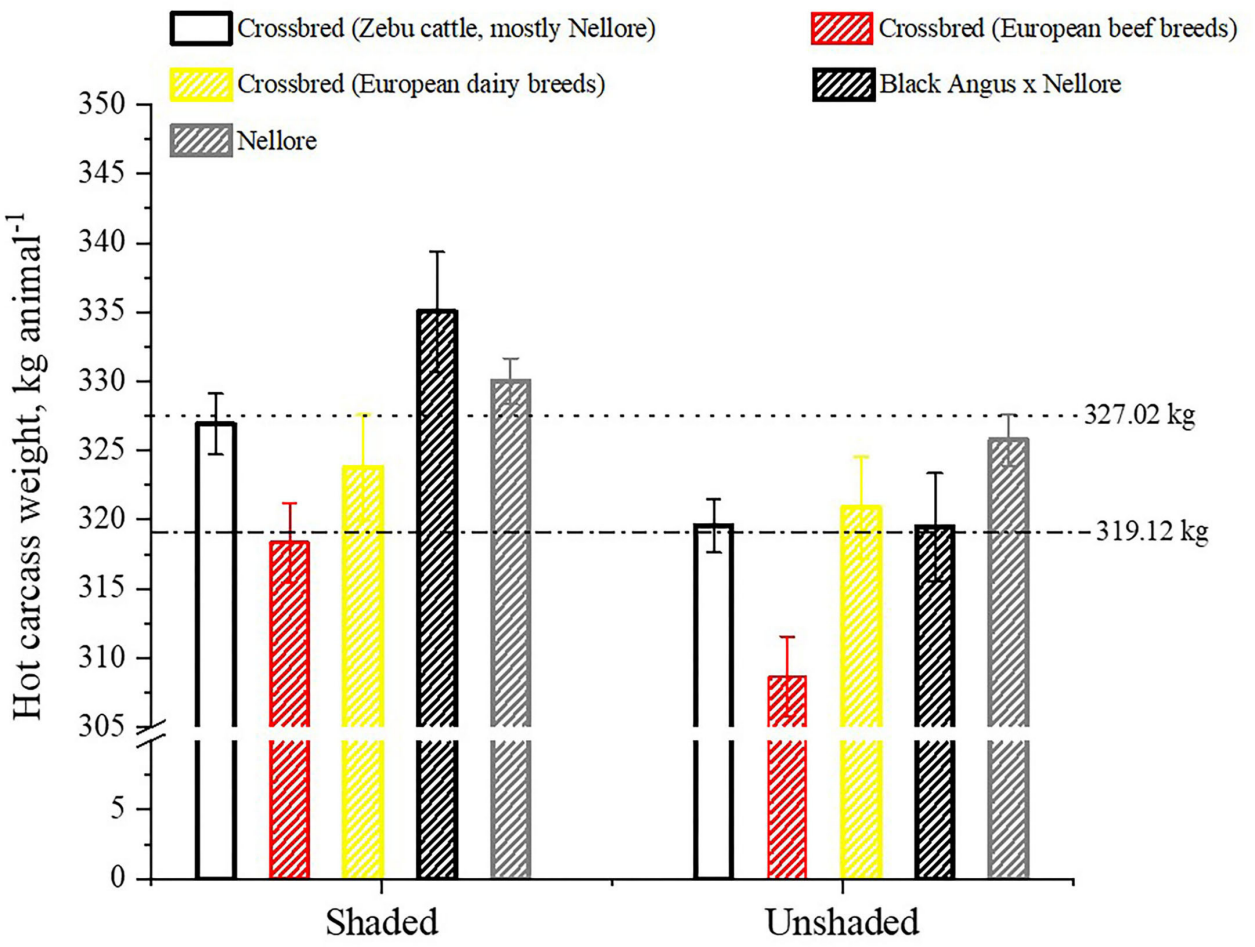
Least square means (±SEM) of hot carcass weight (HCW, kg) of cattle housed in pens with shade and without shade. Dotted line is the least square mean of HCW for the shaded pens; dashed line is the least square mean of HCW for the unshaded pens.

The higher average daily gain of bulls in pens with shade reflected in heavier (*P* = 0.0002) hot carcass weight than those kept in pens without shade (327.02 ± 1.32 vs. 319.12 ± 1.27 kg; Figure 11). Phenotypes of cattle characterized as *B. indicus* Nellore, mean difference for hot carcass weight between cattle in shade and no shade pens was close to 5 kg, while those grouped as crossbred Angus x Nellore, the difference was nearly 15 kg, which suggests that less heat tolerant breeds of cattle can benefit more with heat alleviating strategies. Physiological, metabolic, and behavioral mechanisms of combating heat stress may affect gain efficiency of growing cattle (46). For example, when exposed to high heat load, more blood flows toward the skin to increase heat loss (47), a physiological mechanism that can compromise nutrient absorption and post-absorptive metabolism (48). In this study, a higher level of peripheral vasodilatation may have contributed for the lower growth performance for bullocks in the pens without shade compared to those in pens with shade. In a recent meta-analytical study, Edwards-Callaway et al. (16) also reported that gain efficiency of cattle housed in pens with shade improved by 3.4%, by reflecting mean increments of hot carcass weight of 6 kg. Our study is the first to confirm that use of shade improves gain efficiency even for more heat tolerant breeds of cattle such as *B. indicus* Nellore.

**Figure 12:**
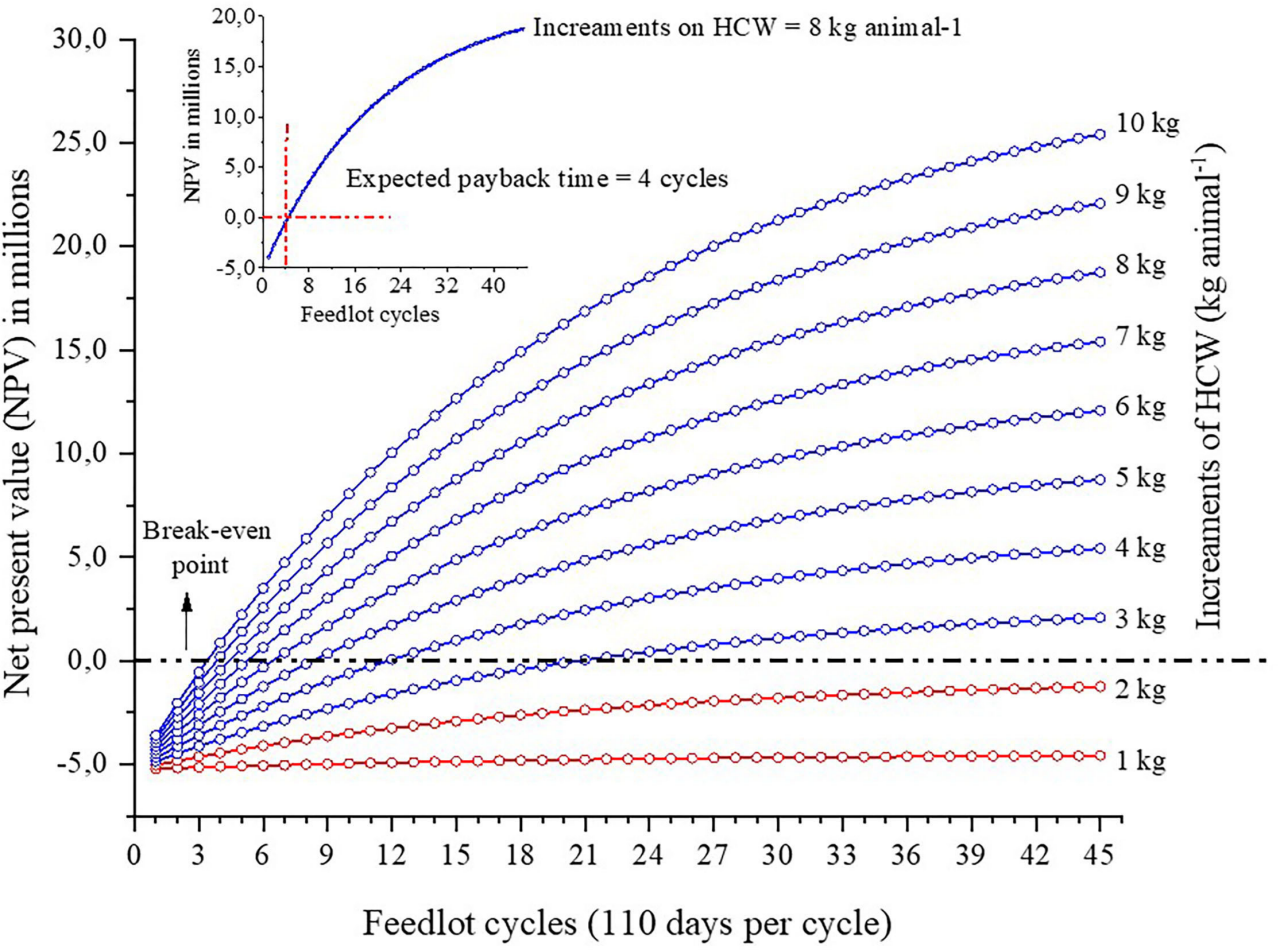
Net present value as a function of number of feedlot cycles for different increments in hot carcass weight. Carcass value at slaughter was taken in November 16, 2020. Dashed line delimits the break-even point; red dotted line represents level of increment for HCW that break-even point is not attained.

The economic feasibility of this study was done on the basis of net present value. The increment in hot carcass weight resulted in a payback time within the first four feedlot cycles (Figure 12). Considering that feedlots may run three feeding cycles per year (or at least two), the payback time for this shade structure, which has an expected lifespan of 15 years, is < 2 years. Also, the economic analysis shows that the break-even point is not reached if increments on hot carcass weight of finished cattle are lower than 2.5 kg animal^−1^. Similarly, during the summer months in Australia, Sullivan et al. (13) reported gain efficiency of Black Angus heifers in pens with shade (using 80%-blockage shade-cloth), increments of AU$ 60 animal^−1^ on carcass value. It is important to note, however, that use of shade cloth will not fully protect animals from direct and diffuse short-wave solar radiation, and have poor durability and life-span (18), especially if installed at locations where heavy wind and rainy conditions exist (19). The shade structure design which was installed in 2019 have been highly resilient to heavy rain (>50 mm hr^−1^) and wind (80 km hr^−1^).

**Figure 13:**
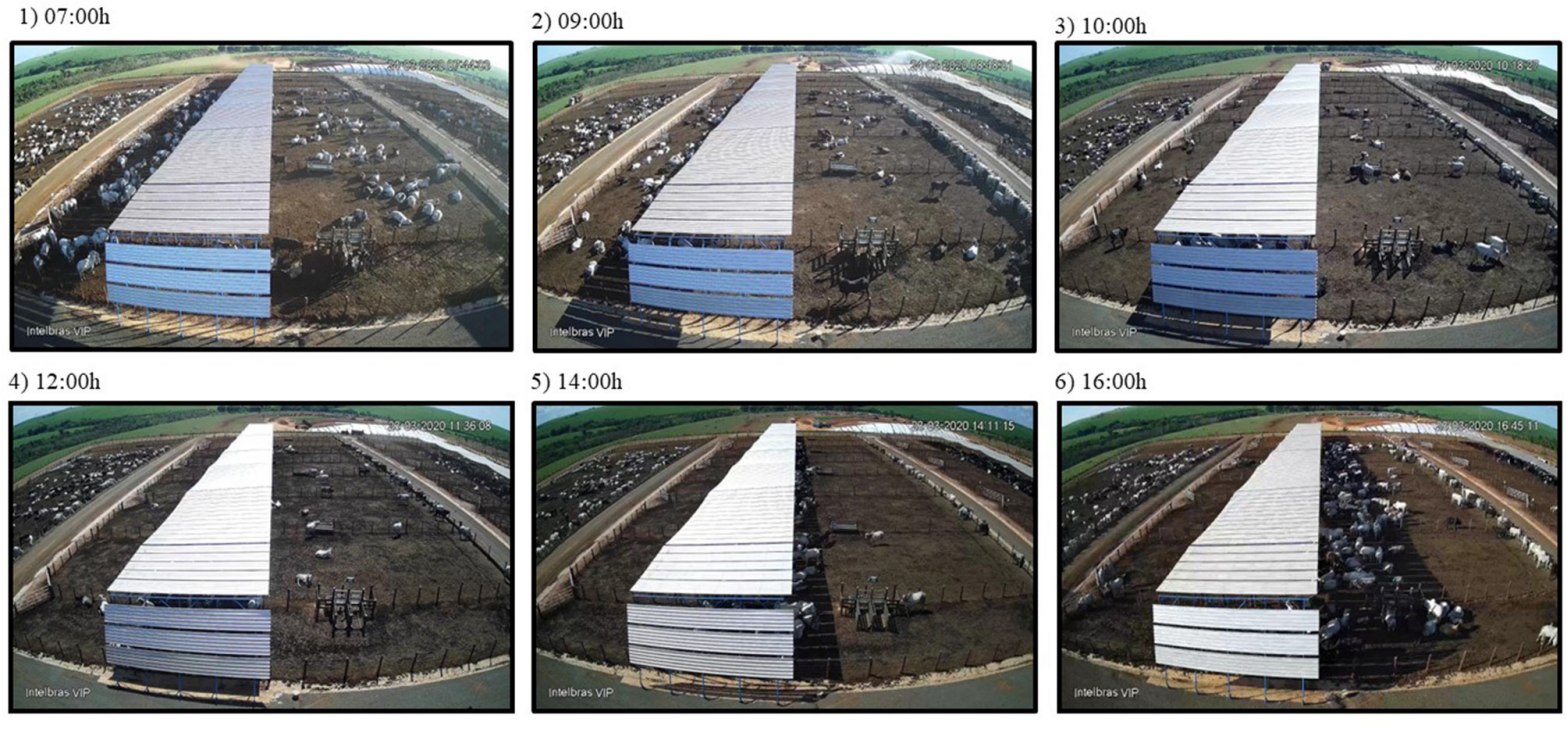
Displacement of shade projection from east to west (20° 31' 25” S; 49° 03' 32” W) at different hours of the day. (March, 20, 2020). Bulls seem to move in the pen following the displacement of the shade. Image source: Courtesy of Agro-Pastoril Paschoal-Campanelli.

The concept of this novel shade design proposed herein challenges the conventional east-west orientation design normally used for shade structures in tropical environments. The later design projects a “static” shade under the roof structure, and consequently becomes causes for cattle congregation, soil compression and mud formation. To alleviate these problems, we propose a combination of rectangular pens with a roof structure-oriented north-south that makes the shade to displace along the east-west direction along the pen (Figure 13). The outcome of using this design motivated cattle to seek shade, resulted in reduced heat stress indicators, decreased water intake and improved feed efficiency. We believe that the economic gains demonstrated from using this novel shade design will promote its use in feedlot operations in tropical climates. More robust economic outcomes however can be obtained through analyses of at least two feedlot cycles in a year, e.g., by taking into account period of the year with more days of moderate cold. At the latitude of the present study (20°S), cold days are likely to occur between May and July. Furthermore, further investigations are also needed to attest if benefits observed in this study can be replicated in other latitudes.

## 4. Conclusions

The following conclusions can be drawn from this study which involved 800 feedlot cattle randomly assigned in pens with and without shade access to determine the effectiveness and economic viability of shade use:

1. The proposed novel shade design combines a roof structure with a north-south orientation, rectangular instead of square pens (length at least three times higher than width), and roof height of at least five meters. This design allows almost 100% of the pen area to be in shade throughout the day.
2. Cattle in pens with shade presented higher average daily gain, reflecting in heavier hot carcass weight (8 kg per animal) compared to cattle with no access to shade.
3. The novel shade design effectively buffers the negative impacts of high radiant heat load on cattle and resists adverse weather impacts as high as 50 mm hr^−1^ precipitation combined with >80 km hr^−1^ wind speed.
4. The expected payback period for the novel shade design was within four finishing cycles (~110 days per cycle), assuming an initial investment of USD$90 per animal to build the structure that lasts 15 years.

## Data availability statement

The raw data supporting the conclusions of this article will be made available by the authors, without undue reservation.

## Ethics statement

All protocols performed in the study were approved by the IACUC of the São Paulo State University, Brazil.

## Author contributions

Conceptualization: AM, RP, GM, and VC. Methodology, validation, and data collection: AM, RP, and GM. Formal analysis: AM and GM. Writing original draft–manuscript: VF. Original draft-figures: AM, GM, and VF. Writing–review and editing: AM, HM, KG, and RP. Visualization: AM, KG, RP, GM, HM, MC, and VF. Supervision: AM and RP. Funding acquisition: AM, VC, and RP. All authors contributed to the article and approved the submitted version.
